# First Stability Characterization for a CZT Detection System in an e^+^e^−^ Collider Environment

**DOI:** 10.3390/s24237562

**Published:** 2024-11-27

**Authors:** Leonardo Abbene, Francesco Artibani, Manuele Bettelli, Antonino Buttacavoli, Fabio Principato, Andrea Zappettini, Massimiliano Bazzi, Giacomo Borghi, Mario Bragadireanu, Michael Cargnelli, Marco Carminati, Alberto Clozza, Francesco Clozza, Luca De Paolis, Raffaele Del Grande, Kamil Dulski, Laura Fabbietti, Carlo Fiorini, Carlo Guaraldo, Mihail Iliescu, Masahiko Iwasaki, Aleksander Khreptak, Simone Manti, Johann Marton, Pawel Moskal, Fabrizio Napolitano, Szymon Niedźwiecki, Hiroaki Ohnishi, Kristian Piscicchia, Yuta Sada, Francesco Sgaramella, Diana Laura Sirghi, Florin Sirghi, Magdalena Skurzok, Michal Silarski, Antonio Spallone, Kairo Toho, Lorenzo Toscano, Marlene Tüchler, Oton Vasquez Doce, Johann Zmeskal, Catalina Curceanu, Alessandro Scordo

**Affiliations:** 1 Dipartimento di Fisica e Chimica—Emilio Segrè, Università di Palermo, Viale Delle Scienze, Edificio 18, 90128 Palermo, Italy; leonardo.abbene@unipa.it (L.A.); antonino.buttacavoli@unipa.it (A.B.); fabio.principato@unipa.it (F.P.); alberto.clozza@lnf.infn.it (A.C.); francesco.clozza@lnf.infn.it (F.C.); luca.depaolis@lnf.infn.it (L.D.P.); 2 Laboratori Nazionali di Frascati, INFN, Via E. Fermi 54, 00044 Frascati, Italy; bazzi@lnf.infn.it (M.B.); raffaele.del-grande@tum.de (R.D.G.); guaraldo@lnf.infn.it (C.G.); mihai.iliescu@lnf.infn.it (M.I.); simone.manti@lnf.infn.it (S.M.); fabrizio.napolitano@lnf.infn.it (F.N.); kristian.piscicchia@centrofermi.it (K.P.); francesco.sgaramella@lnf.infn.it (F.S.); diana.laura.sirghi@lnf.infn.it (D.L.S.); fsirghi@lnf.infn.it (F.S.); antonio.spallone@lnf.infn.it (A.S.); oton.vazquez.doce@cern.ch (O.V.D.); catalina.curceanu@lnf.infn.it (C.C.); alessandro.scordo@lnf.infn.it (A.S.); 3 Dipartimento di Matematica e Fisica, Università di Roma Tre, Via della Vasca Navale, 84, 00146 Roma, Italy; 4 Istituto Materiali per l’Elettronica e il Magnetismo, Consiglio Nazionale delle Ricerche, Parco Area delle Scienze 37/A, 43124 Parma, Italy; manuele.bettelli@imem.cnr.it (M.B.); andrea.zappettini@imem.cnr.it (A.Z.); 5 Politecnico di Milano, Dipartimento di Elettronica, Informazione e Bioingegneria, Via Giuseppe Ponzio 34, 20133 Milano, Italy; giacomo.borghi@polimi.it (G.B.); marco1.carminati@polimi.it (M.C.); carlo.fiorini@polimi.it (C.F.);; 6 INFN Sezione di Milano, Via Celoria 16, 20133 Milano, Italy; 7 Horia Hulubei National Institute of Physics and Nuclear Engineering (IFIN-HH), No. 30, Reactorului Street, Magurele, 077125 Ilfov, Romania; mario.bragadireanu@nipne.ro; 8 Stefan-Meyer-Institut für Subatomare Physik, Dominikanerbastei 16, 1010 Wien, Austriajohann.marton@oeaw.ac.at (J.M.); marlene.tuechler@oeaw.ac.at (M.T.); johann.zmeskal@oeaw.ac.at (J.Z.); 9 Physik Department E62, Technische Universität München, James-Franck-Straße 1, 85748 Garching, Germany; laura.fabbietti@ph.tum.de; 10 Institute of Physical and Chemical Research, RIKEN, 2-1 Hirosawa, Wako, Saitama 351-0198, Japan; masa@riken.jp; 11 Faculty of Physics, Astronomy, and Applied Computer Science, Jagiellonian University, Łojasiewicza 11, 30-348 Krakow, Poland; aleksander.khreptak@lnf.infn.it (A.K.); p.moskal@uj.edu.pl (P.M.); szymon.niedzwiecki@uj.edu.pl (S.N.); magdalena.skurzok@uj.edu.pl (M.S.); michal.silarski@uj.edu.pl (M.S.); 12 Centre for Theranostics, Jagiellonian University, Kopernika 40, 31-501 Krakow, Poland; 13 Research Center for Accelerator and Radioisotope Science (RARIS), Tohoku University, 1-2-1 Mikamine, Taihaku-kun, Sendai 982-0826, Japan; ohnishi@lns.tohoku.ac.jp (H.O.); toho.kairo.t4@dc.tohoku.ac.jp (K.T.); 14 Centro Ricerche Enrico Fermi—Museo Storico della Fisica e Centro Studi e Ricerche “Enrico Fermi”, Via Panisperna 89A, 00184 Roma, Italy

**Keywords:** CZT detector, X-ray detector, kaonic atoms

## Abstract

The SIDDHARTA-2 collaboration has developed a novel X-ray detection system based on cadmium-zinc-telluride (CZT, CdZnTe), marking the first application of this technology at the DAΦNE electron-positron collider at INFN-LNF. This work aims to demonstrate the stability of the detectors’ performance in terms of linearity and resolution over short and long periods, thereby establishing their suitability for precise spectroscopic measurements within a collider environment. A reference calibration spectrum is presented in association with findings from assessments of linearity and resolution stability. Additionally, this study introduces a validated model of the response function of the detector. The relative deviations from the nominal values for the source transitions, obtained by fitting the entire spectrum with a background function and the previously introduced response function, are reported. Finally, a comparison of the calibration performance with and without beams circulating in the collider’s rings is presented. These promising results pave the way for applying CZT detectors in kaonic atom studies and, more generally, in particle and nuclear physics spectroscopy.

## 1. Introduction

Cadmium-Zinc-Telluride (CZT) is an attractive compound semiconductor for room-temperature X-ray detectors, offering excellent energy and timing resolutions across a broad energy range [1]. As a high-Z material, CZT allows for a high detection efficiency up to hundreds of keV. Additionally, its large bandgap ensures ideal performance for X-ray and γ-ray spectroscopy at room temperature. These unique properties, coupled with recent advances in crystal growth techniques and electrical contact technologies, have established CZT as one of the most promising materials for semiconductor-based room-temperature X-ray spectroscopy [2,3,4,5,6]. Building on these advantages, the SIDDHARTA-2 (Silicon Drift Detectors for Hadronic Atom Research by Timing Application) collaboration has developed a novel CZT-based detection system for X-ray spectroscopy at the DAΦNE collider [7,8], laying the groundwork for the application of this semiconductor technology to nuclear and sub-nuclear physics research.

The collaboration leverages the unique low-energy kaon beam produced by the DAΦNE collider at the Laboratori Nazionali di Frascati (LNF) to conduct research on kaonic atoms [9,10,11,12]. Kaonic atom spectroscopy [13,14,15] holds particular importance in the fields of particle and nuclear physics [16,17], with additional applications in exotic atoms cascade models [18] and in astrophysics [19,20]. Through the spectroscopy of kaonic atoms, two key observables related to low-energy strong interactions can be measured from the kaons’ transitions between two atomic orbitals: a shift (ϵ) in energy relative to the purely electromagnetic process and an intrinsic width (Γ) due to the limited lifetime of the state assigned to the level, which causes a broadening of the spectral line. These observables serve as essential inputs for theoretical models of low-energy strong interactions [16,17].

Focusing on kaonic atoms heavier than hydrogen and deuterium, a series of experiments conducted between the 1970s and 1980s produced an extensive set of measurements of shifts and widths for many kaonic atoms across the periodic table [21,22]. However, recent experiments [23] have reported different values for some atoms, indicating that earlier results may be unreliable and raising doubts about the current understanding of kaon interactions with multiple nucleons (K–multiN interaction). Moreover, with modern technologies, higher precision can be achieved, allowing stronger constraints to be placed on phenomenological models [16,17].

Specifically, intermediate-mass kaonic atoms, such as kaonic aluminum, copper, carbon, and sulfur, have exhibited inconsistent measurement results and significant uncertainties. These atoms’ transitions are characterized by intrinsic shifts and widths from a few eV to tens of keV depending on the principal quantum number of the level and the nucleus. All previous measurements are reported in [21].

To perform new measurements of intermediate-mass kaonic atom systems, the SIDDHARTA-2 collaboration developed a new detection system based on the promising CZT compound semiconductor. Since CZT detectors have never been previously used in accelerator-based experiments, their behavior under high and variable background conditions has not been assessed. Thus, the collaboration already performed a first series of tests demonstrating the detector’s suitability in terms of resolution, efficiency, and timing [6,7,8].

This paper reports the results of investigations into short (hours) and long (months) term stabilities of the detector response, as well as the possible impact of the DAΦNE beam on the CZT performance. In kaonic atoms experiments, one of the most critical contributions to systematic errors is due to miscalibration of the detector, as previously investigated by the SIDDHARTA-2 collaboration [24,25,26].

The tests presented in this work were carried out with an improved version of the apparatus compared to the previous setup: first, eight CZT detectors were used, with a maximum active area of 15.6 cm^2^; second, the detectors were placed closer to the DAΦNE interaction region (IR) to assess the performance in higher background conditions.

In Section 2, the accelerator, detectors, and procedures adopted during the various runs are described. Section 3 provides a summary of the data analysis strategy and the obtained results. Finally, in Section 4, a discussion of the results is reported.

## 2. Materials and Methods

### 2.1. The DAΦNE Collider

The experiment was conducted at the e^+^e^−^ DAΦNE collider [27,28,29] at the Laboratori Nazionali di Frascati (LNF) of the INFN (Istituto Nazionale di Fisica Nucleare). This machine operates at a center-of-mass energy of 1.020 GeV, where the cross-section for e^+^e^−^ interactions is dominated by ϕ resonance production. This machine is unique for kaonic atom studies because the ϕ resonance decays into a pair of low-momentum charged kaons with a branching ratio of 48%. Such low-energy kaons can be effectively stopped in a target material, allowing them to form kaonic atoms suitable for spectroscopy.

In the DAΦNE collider, the experimental background induced by the machine is primarily due to the Touschek effect, which occurs when dense beams are utilized at relatively low energies to achieve maximum luminosity [30]. This effect generates background due to off-energy particles resulting from electromagnetic interactions within a single bunch. As a result, a substantial number of off-momentum particles are produced. Those particles that have a momentum significantly deviating from the nominal value are lost in the focusing magnets, before reaching the interaction point and exit the beam line. This leads to a pronounced electromagnetic background, particularly in the area where the CZT detector is placed.

Typical experiments carried out at the DAΦNE collider require extended data-taking periods of several months, during which the machine is frequently switched off and its conditions (currents, temperatures, vibrations, mechanical noise, etc.) can significantly vary. It is known that such variations had a non-negligible influence on the Silicon Drift Detectors (SDDs) used by the collaboration in other measurements [24,25,26]. Careful investigations of similar effects on CZT detectors are then mandatory for future measurement campaigns.

### 2.2. CZT Detection System

The CZT detection system used in this work consists of eight 13mm×15mm×5mm quasi-hemispherical CZT detectors, enclosed in a thin aluminum box with a 0.27 μm thick aluminum window. These detectors were provided by REDLEN Technologies (Canada) and will be referred to as “commercial detectors”. During one of the tests, four of the REDLEN detectors were replaced by three 10mm×10mm×5mm quasi-hemispherical CZT detectors provided by IMEM-CNR in Parma (Italy), which were used for the first time at the DAΦNE collider. The detectors are coupled to custom front-end electronics based on analog charge-sensitive preamplifiers (CSPs), developed by the research group of the University of Palermo (Italy). The CSPs are characterized by a resistive-feedback circuit with exponential decay and a time constant of 100 μs and nominal equivalent noise charge (ENC) of 100 electrons (equivalent to 1 keV FWHM for CZT detectors). The output signals from CSPs are processed by digital electronics based on custom pulse processing techniques. The digital electronics consists of a commercial digitizer with 64 channels (VX2740—16 bit 125 MS/s, CAEN S.p.A., Viareggio, Italy; http://www.caen.it), driven by an original firmware developed at Dipartimento di Fisica e Chimica (DiFC) Emilio Segré of Palermo University [31,32]. Both acquisition and analysis are controlled by a PC.

The digital system performs online acquisition of the CSP output waveforms from all eight detectors, in self-trigger mode (i.e., each detector/CSP channel is characterized by a proper asynchronous trigger). The acquired data from each channel are a list of CSP waveforms, termed snapshot waveforms (SWs). Each snapshot contains a single CSP output pulse with the related arrival time. The CSP pulse is centered on the snapshot and its time width is termed snapshot time (ST). A pulse is accepted if it is neither preceded nor followed by another pulse in the ST/2 time windows, acting as a pile-up rejection (PUR), i.e., avoiding the presence of the effects of pile-up in the energy spectra. The ST parameter can be selected by the user.

Each pulse of a single snapshot is coupled to its arrival time, measured online by using the single delay line (SDL) shaping technique, i.e., by using a differentiation operation in the time domain. The time width of the SDL pulses (equal to the sum of the peaking time and the delay time) represents the dead time of the detection process (paralyzable dead time).

The arrival time of each event is calculated through the ARC (Amplitude and Rise time Compensation) timing marker (at the leading edge of the SDL pulses), necessary to reduce the effects of time jitters and amplitude/rise time walks. The timing resolution is less than 10 ns. Therefore, the online acquisition can provide the arrival time and the CSP waveform for each pulse and the input counting rate (ICR). In our case, we used, for pulse detection, a delay time of 150 ns, producing a paralyzable dead time of 290 ns (maximum ICR of 6.7 Mcps).

The CSP output pulses of the SWs are offline processed with dedicated analysis. The height (i.e., the photon energy) of the pulses is calculated after trapezoidal shaping. A C++-based program was developed to set the parameters of the digitizer (acquisition time, channels, etc.) for the acquisition of the SWs. Another program (Labview) was developed for offline analysis of the pulses of the SWs and to generate the energy spectra. Each SW was acquired with a snapshot time (ST) of 10 μs, producing a dead time for the output counting rate (OCR) equal to ST-290 ns. This allows a throughput (i.e., the OCR/ICR ratio) in the energy spectra of 99% up to 1500 cps, which is the maximum measured ICR in the detectors with the beam on. As a reference, with the beam off a lower (50 cps) rate is registered on CZT. A trapezoidal shaping, with a peaking time of 1400 ns and 100 ns of flat top, was used for offline pulse shaping. A more detailed description of the readout logic and waveform analysis can be found in [6].

The electronic components are enclosed in the aforementioned aluminum box and are identical for all the detectors. To stabilize the temperature of the box, a FRYKA DLK 402 recirculating chiller, operating at a temperature of 15 °C, was placed on its lower side. The lateral sides of the box were surrounded by a lead shielding to reduce the intense radiative background caused by particle losses at the last focusing quadrupole near the IR at DAΦNE.

**Figure 1:**
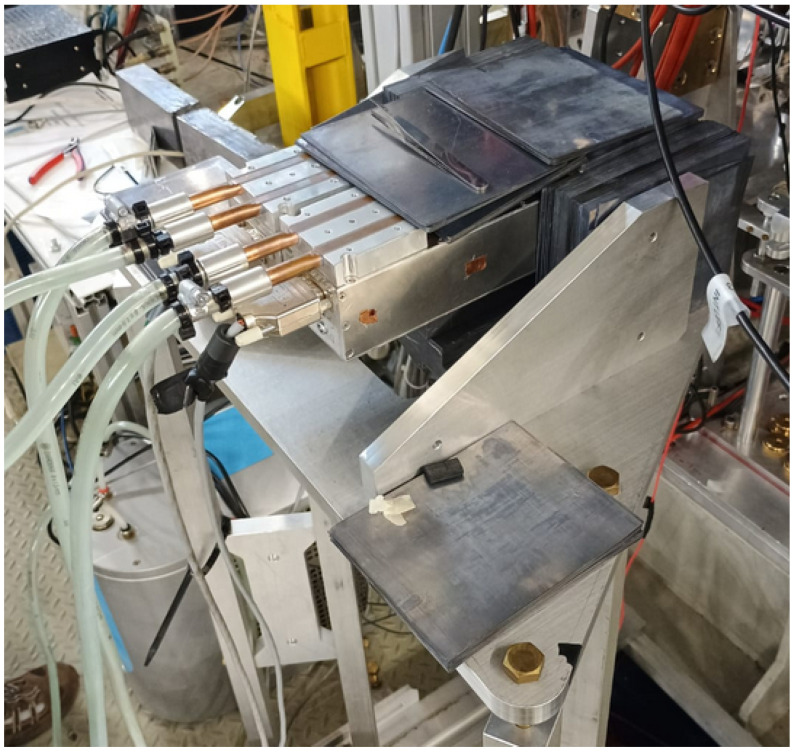
Photo of the CZT detection system placed in the DAΦNE interaction region. The detectors in the aluminum box, surrounded by the lead foils shielding, can be seen at the center. On the left-hand side, over the box, the chiller system can be found. The detector lays on a special support that allows it to change its position to the beam pipe.

A photo of the detection system can be found in Figure 1.

The signals coming from the CZT are finally sent to a CAEN DT5780 and a V2740 digitizer, driven by an original firmware.

### 2.3. Calibrations

The radioactive source used for calibrations was ^152^Eu. Following LARA (Library for gamma and Alpha emissions, http://www.lnhb.fr/Laraweb/, accessed on 10 September 2024), in Table 1, the expected energy lines coming from an ^152^Eu source in the region inspected by the detector are reported.

During the data acquisition for the SIDDHARTA-2 experiment at the DAΦNE collider, the CZT detector also collected data over several months. Initially, the detector underwent an extended setup optimization phase, which included adjustments of the high voltage (HV) and the detector’s position. In this study, we report on calibrations conducted with a consistent high voltage of 900 V applied to the detector. A list of the calibrations used in this work can be found in Table 2.

**Figure 2:**
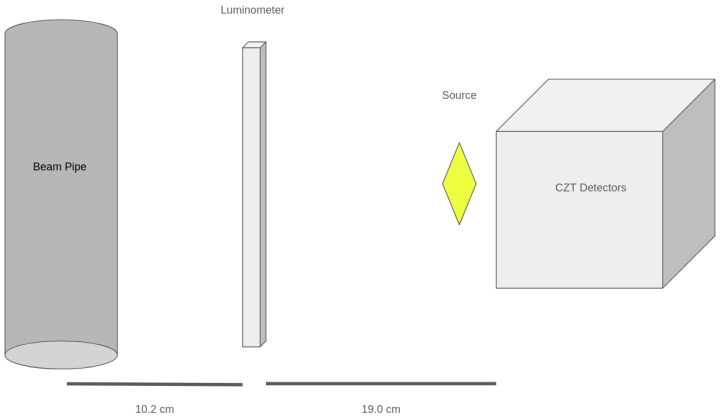
Schematic view of the setup during the run with the source.

To evaluate the possible effect of the circulating beams on the CZT’s response function, a test run with beams on and the use of the radioactive source was conducted. The detection system was placed 25 cm from the DAΦNE collider Interaction Point (IP), with a plastic scintillator, read by two PMTs, positioned 10.2 cm from the IP, placed between the detector and the IP to serve as a luminosity monitor for the experiment [8,33]. Figure 2 shows a schematic view of the experimental setup.

The run lasted approximately 13 h.

## 3. Analysis and Results

### 3.1. Peak Fit Function

CZT detectors at room temperature exhibit a non-negligible tailing effect that must be properly characterized.

The two main contributions to the spectral peaks are:

1. Gaussian function. This represents the energy dispersion caused by the intrinsic resolution of the detector. It can be described as a Normal distribution with three parameters: the mean value, the dispersion coefficient, or standard deviation, and a normalization factor.
2. Tail function. The Gaussian function alone cannot adequately describe the spectral peaks. Events with incomplete charge collection in the semiconductor must also be parameterized alongside those with complete charge collection. In [34], an in-depth study of the tailing effect in semiconductor X-ray spectroscopy demonstrated that an exponential function best describes the tail distribution. Additionally, ref. [35], which focuses specifically on CZT detectors, shows that such a function effectively fits the errors in charge collection.

In this work, the tails were modeled using the function suggested in the previously cited articles [34,35]. The fitting function used for a single peak is:

(1) $$f_{peak}\left(x\right)=N\;\times \;\exp\left(-\frac{x-\mu }{2\sigma^{2}}\right)\;+\;\epsilon \;\times N\;\times \exp\left(\frac{x-\mu }{\beta \sigma }\right)\;\times \;\mathrm{erfc}\left(\frac{x-\mu }{\sqrt{2}\sigma }\;+\;\frac{1}{\sqrt{2}\beta }\right),$$

where *erfc* denotes the complementary error function, which serves to truncate the exponential tail before the Gaussian mean.

**Figure 3:**
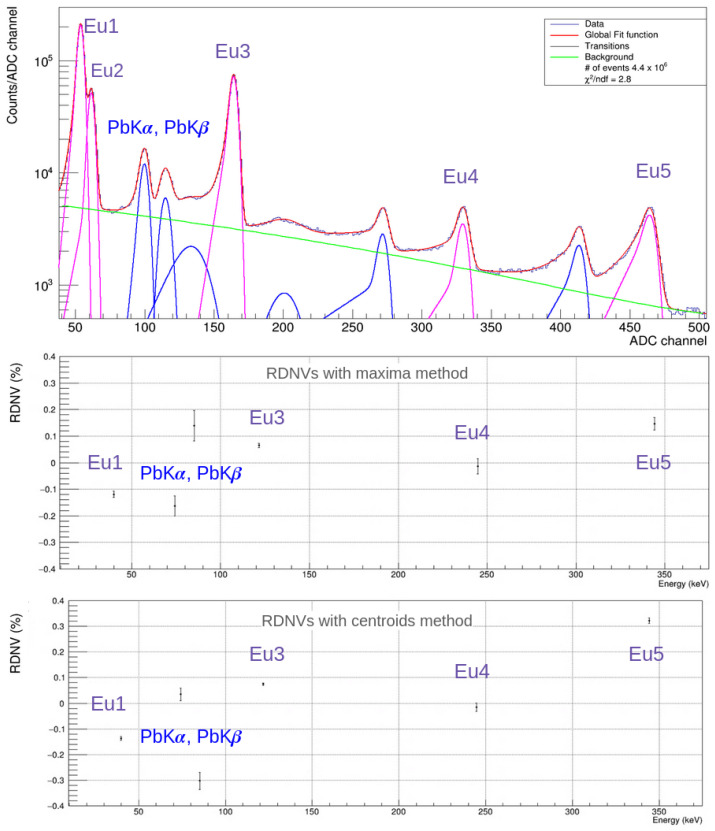
Top: Spectrum collected by one of the detectors in the run held from the 5 to the 6 May 2024. The radioactive source’s transition peaks are highlighted in purple, while the peaks resulting from particle interactions with the shielding are shown in blue. Bottom: RDNVs obtained after calibrating the spectrum.

In Equation (1), there are five free parameters: *N* represents the normalization factor of the Gaussian function, μ is the mean of the Gaussian function, i.e., the center of the peak, σ stands for the standard deviation of the Gaussian function, ϵ represents the fraction of the height of the tail relative to the Gaussian, and β represents the width of the tail function. The function effectively describes the tailing effect, as shown in the spectrum in Figure 3.

### 3.2. Fit of Calibration Spectra

A typical spectrum from one of the eight CZT channels is displayed in the upper part of Figure 3. In this spectrum, the background is modeled as a second-order polynomial. Several peaks are identified: five originate from the Europium source (labeled as described in Table 1), and four result from interactions between the lead shielding surrounding the detectors and the source’s γ-rays. After calibration, the first two peaks, at about 100 ADC and 115 ADC, correspond to 75 keV and 85 keV, respectively. These two peaks result from the de-excitation of lead in the shielding following interactions with high-energy photons from Eu decays. The lead $K_{\alpha 1}$, $K_{\alpha 2}$, $K_{\beta 1}$, $K_{\beta 2}$, and $K_{\beta 3}$ transitions have energies of 74.969 keV, 72.805 keV, 84.938 keV, 87.300 keV, and 84.450 keV, respectively. Weighted means of these transitions, based on the corresponding expected yields as reported in [36], were used as the reference energy for these two peaks. The two peaks at higher ADC values (approximately 270 and 410, corresponding to roughly 200 keV and 300 keV after calibration) were identified as backscattering peaks resulting from the Compton scattering in lead. This process occurs when high-energy γ-rays from Europium decay interact with the shielding through Compton backscattering and are subsequently captured by the detector.

For calibration, three of the five visible peaks from Europium were selected: Eu1, Eu3, and Eu4. This selection was made since the Eu2 peak is not consistently detectable by all detectors, as it lies only 5 keV away from the more intense Eu1 peak, and the resolution of some detectors is a few percentage points worse, especially at lower energies (<50 keV), compared to the performance detailed in Table 3. This limitation arises from high-noise electrical contacts in certain detectors and the presence of inclusions within the CZT crystals, which act as charge traps. These issues underscore that fabricating high-resolution CZT detectors remains a critical challenge. The Eu5 peak, which exhibits significant tailing and distortion effects, was nonetheless fitted and chosen as a cross-check for calibration.

To assess the quality of the calibration procedure, the Relative Deviation from Nominal Value (RDNV) has been introduced, defined as:

(2) $$\mathrm{RDNV}\left(\%\right)=\frac{\left(E_{true}-E_{meas}\right)}{E_{true}}.$$

In the lower part of Figure 3, the RDNVs are reported for two different calibration methods.

According to studies conducted on Cadmium-Telluride (CdTe), another semiconductor with a significant tailing effect [37], the reference energy (or ADC) value extracted from the fit should be the maximum of the function $f_{peaks}$, rather than the mean of the Gaussian component. It was demonstrated that, while the maxima and centroids of the Gaussians are closely aligned for peaks with minor tailing (those in the region of interest), using the maximum avoids potential systematics introduced by the pronounced tailing effect observed in these detectors at higher energies (300 to 500 keV). This behavior was also confirmed for the CZT detection system. For example, Figure 3 presents the resulting RDNVs when calibrating one of the detectors using both the centroid and maximum methods. In the figure, it is evident that the RDNVs for the Europium peaks below 300 keV are nearly identical, while at 344 keV, where tailing effects are stronger, the residual using the centroid method is twice that of the maximum method. This trend was observed across all detectors, and therefore, in the subsequent studies presented in this article, the maximum method was consistently applied.

For the two lead fluorescence peaks, the RDNVs calculated using the two methods differ. This RDNV discrepancy arises from the convolution applied during peak fitting. In this scenario, the tail tends to incorporate the lower-yield transitions (Pb Kα2 for Pb Kα and Pb Kβ2, Pb Kβ3 for Pb Kβ), creating a broader profile that shifts the maxima further from the centroids. In contrast, the Europium peaks, being farther from other transitions, have tails that only indicate incomplete charge collection, so this effect does not arise.

Similarly, the resulting resolution is not that of the Gaussian but rather the FWHM of $f_{peaks}$, which also accounts for the tailing effect.

### 3.3. Short-Term Stability

In an environment like the DAΦNE collider, studying the CZT system stability is crucial due to potential sources of electronic instability. This work presents a stability analysis conducted over a single run, followed by an evaluation of stability over a longer period across multiple runs. These findings are essential to confirm the reliability of the detectors and the data collected during physics runs, demonstrating that the CZT detection system can achieve the precision necessary for measuring kaonic atom observables.

**Figure 4:**
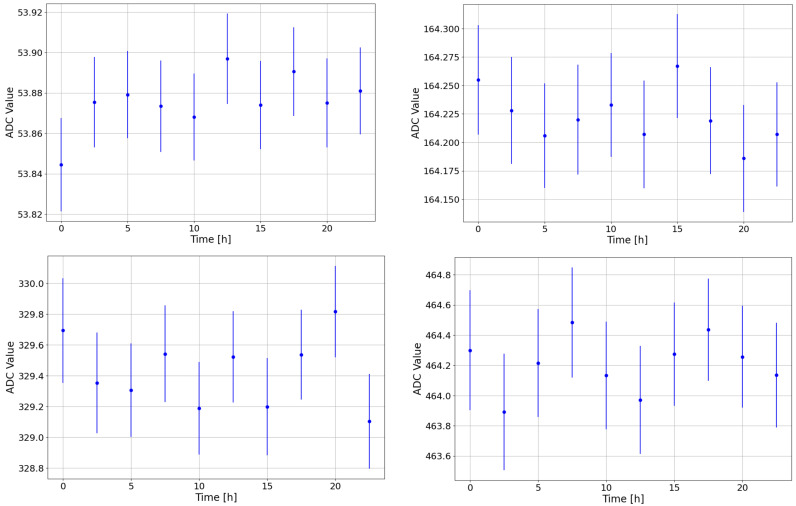
Values of the ADC channel of the ^152^Eu peaks obtained from one of the detectors across the different datasets for the peak at 40 keV (**top left**), at 121 keV (**top right**), at 244 keV (**bottom left**), and at 344 keV (**bottom right**).

The stability during a single calibration run was examined by dividing the longest calibration run, conducted between the 6 and 7 May 2024, into ten 2.5-h datasets. Each dataset was fitted using the previously described procedure. Figure 4 presents the ADC values of the peaks for one of the detectors, as a reference.

The plot demonstrates that the detector is extremely stable during a 24-h run, exhibiting a dispersion of O ($10^{-3}$ keV) across the 2.5-h datasets. This study confirms the excellent stability of both detectors and electronics.

### 3.4. Long-Term Stability

**Figure 5:**
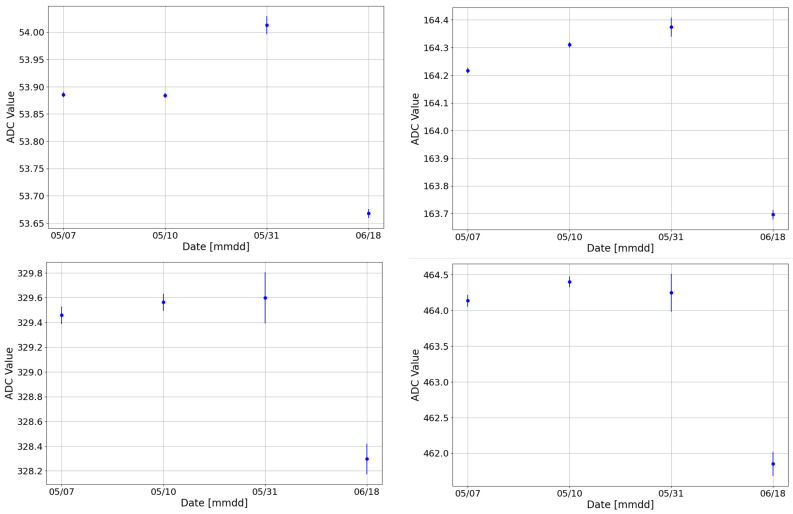
Position of the ADC maxima for the ^152^Eu peaks at 40 keV (**top left**), 121 keV (**top right**), 244 keV (**bottom left**), and 344 keV (**bottom right**) for different dates following the calibrations of one of the detectors.

It is also essential to check that CZT performance and calibration parameters do not change after DAΦNE switch off or, more generally, over a long period. The long-term stability was then studied by analyzing the dataset in Table 2. In Figure 5, the ADC values from the performed calibrations are shown. The ADC channels of the Europium peaks resulted in varying by less than 1%, confirming the good stability of this system. Some of the ADC values coming from data collected in different runs resulted to be incompatible with each other: this behavior was expected, due to the aforementioned instability in DAΦNE (end of Section 2.1), also observed with the SDDs detectors used by the collaboration in [26].

**Figure 6:**
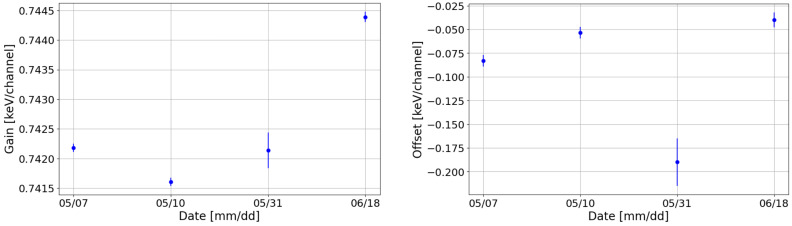
Gains and offsets values for the different calibration runs.

Examining the gains and offsets obtained after the fit for the same channel, as shown in Figure 6, the slight decrease in the ADC values between the run on the 31 May 2024 and that on the 18 June 2024 results in a small increase in the gain (3‰).

**Figure 7:**
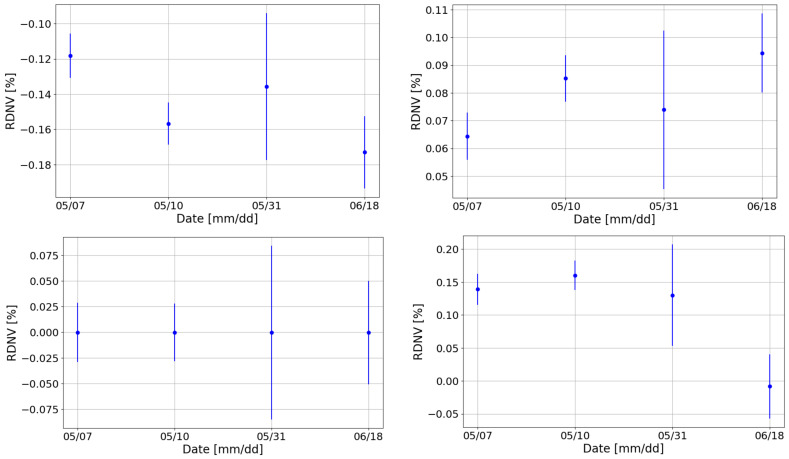
RDNVs of the peaks for one of the detectors obtained after calibrations on different dates for the peak at 40 keV (**top left**), 121 keV (**top right**), 244 keV (**bottom left**), and 344 keV (**bottom right**).

Overall, the detectors exhibited excellent performance: all RDNVs after calibration were found to be within 5‰, also for the 344 keV peak, which was excluded from the calibration process and is particularly sensitive to tailing effects, small fluctuations, and potential miscalibrations. Figure 7 displays the relative RDNVs (Equation (2)) for one of the detectors across multiple calibration instances.

To account for the strong tailing effect, particularly in high-energy peaks, as mentioned previously, the resolution was estimated by evaluating the Full Width at Half Maximum (FWHM) for each peak. As an example, in Table 3, the FWHM values for one of the detectors across different calibrations are reported.

From the table, it is evident that the resolution does not significantly degrade even after months of data taking, confirming the stability of the entire apparatus over more than a month.

**Figure 8:**
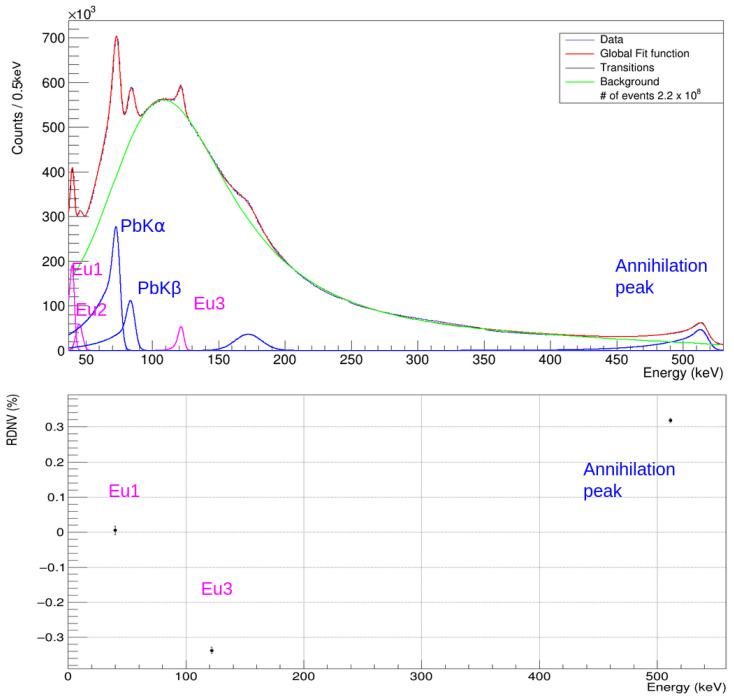
Top: Fit of the spectrum acquired with the commercial detectors during the data taking with the source and beam on. The radioactive source’s transition peaks are highlighted in purple, while the peaks resulting from particle interactions with the shielding are shown in blue. Bottom: relative RDNVs of the ^152^Eu peaks at 40 keV and 121 keV, and for the 511 keV peaks.

Finally, the performance of the detectors during a run with the beam on and the radioactive source was studied. After calibrating the spectrum of each CZT detector, the final spectrum, which represents their combined sum, is obtained. As an example, we present in Figure 8 (top) the spectrum collected for the REDLEN detectors calibrated using the results from the calibration conducted between the 6 and 7 May 2024. The background in DAΦNE was modeled as:

(3) $$f_{bkg}\left(x\right)=a+b\cdot x+c\cdot \exp\left(d\cdot x\right)+\mathrm{erfc}\left(\frac{e-x}{f}\right).$$

The exponential behavior results from the predominant background generated by photons and electrons undergoing Compton scattering. As these particles interact with the air and materials between the beam pipe and the detector, they produce a considerable number of secondary particles with lower energies. The component described by the complementary error function accounts for a cutoff at lower energies resulting from the presence of shielding and electronics. In addition to these two components, a linear function representing the electronic baseline was also included.

The most prominent peaks are those originating from lead fluorescence (Kα and Kβ) in the shielding, along with the peak around 200 keV, attributed to the backscattering processes described earlier. The peaks corresponding to Eu1, Eu2, and Eu3 from the source’s decays are also significant against the background. Lastly, a peak at 511 keV was observed, which is associated with e^+^e^−^ annihilation processes.

In Figure 8, (bottom), the RDNVs obtained from the fit for the Eu1, Eu3, and annihilation peaks are reported.

The RDNVs were found to be O ($10^{-3}$) keV across a wide range (from 40 to 511 keV). The FWHM values obtained from the fit of the summed spectrum are 13.7% at 40 keV and 4.5% at 121 keV, resulting from the convolution of the contribution from each detector. The worsening of the total FWHM at 40 keV is driven by the non-optimal performance of one of the detectors at low energies; on the contrary, the FWHM at 121 keV is compatible with the individual ones, confirming how the influence of the circulating beams on the final resolution is negligible.

The obtained RDNVs can also be interpreted as a preliminary estimation of the systematic errors that may affect the detectors in future runs, serving as an important input for the forthcoming kaonic atoms measurements with CZT.

## 4. Discussion

The studies presented in this article demonstrate that the new CZT detection system developed by the SIDDHARTA-2 collaboration is ideal for X-ray measurements in an environment such as the DAΦNE collider, making it an excellent choice for applications in kaonic atom spectroscopy.

The short-term stability investigation confirmed that possible effects due to slight changes in the operational conditions of DAΦNE (currents, temperature, etc.) during a single data taking are minimal and manageable.

The long-term stability study demonstrated that the system remains stable within a month even after turning off the apparatus and machine and after replacing certain detectors, indicating the robustness of the electronics and the hardware and software data acquisition systems. This finding is particularly significant, as it confirms that a single extended calibration every two to three weeks, or even once a month, is sufficient to accurately control systematics and obtain precise results on kaonic atom observables.

Overall, the stability analysis conducted in this work underscores the reliability of the upcoming results from extended data collection periods. Furthermore, this work proved how individual calibrations performed off-beam can be successfully applied to data acquired with circulating beams, establishing an effective calibration method for future physics runs. It also illustrates that small fluctuations over months, due to environmental factors, can be effectively managed with this approach. Moreover, the high quality of such a calibration procedure is also confirmed by the resolutions obtained from the fit of the summed spectra, showing no significative peak broadening due to miscalibration.

Finally, we demonstrated the reliability of a detector equipped with a dedicated readout system capable of operating at rates in a high-background environment, up to 100 kcps, foreseeing new applications in other accelerators, such as J-PARC.

This test, along with previous studies [6,7,8], represents the final assessment of the detector, as it provides definitive confirmations of the reliability of this detection system within a collider environment and opens new possibilities for intermediate-mass kaonic atoms measurements.

## Figures and Tables

**Table 1:** Transition energies of ^152^Eu in the CZT domain.

Energy (keV)	Intensity (%)	Type	Origin	ID ^1^
40.1186 (-)	37.7 (5)	X $K_{\alpha 1}$	Sm	Eu1
121.7817 (3)	28.41 (13)	γ	^152^Sm	Eu3
344.2785 (12)	26.59 (12)	γ	^152^Gd	Eu5
39.5229 (-)	20.8 (3)	X $K_{\alpha 2}$	Sm	Eu1
45.4777 (-)	11.78 (19)	X $K_{\beta 1}$	Sm	Eu2
244.6974 (8)	7.55 (4)	γ	^152^Sm	Eu4
46.6977 (-)	3.04 (8)	X $K_{\beta 2}$	Sm	Eu2

^1^ The transitions used in this work were labeled in this way. For Eu1 and Eu2, the mean of the two transitions weighted with the intensity was considered as reference energy.

**Table 2:** List of the calibration runs presented in the paper.

Date	Source	Detectors	Duration	Cumulative Luminosity Delivered from the Start of the Run (6 February 2024)	DAΦNE Status
8–9 April 2024	^152^Eu	4 commercial + 3 IMEM-CNR	13 h	374 pb^−1^	Active
6–7 May 2024	^152^Eu	4 commercial + 3 IMEM-CNR	25 h	541 pb^−1^	Inactive
9–10 May 2024	^152^Eu	8 commercial	20 h	541 pb^−1^	Inactive
31 May 2024	^152^Eu	8 commercial	1 h	593 pb^−1^	Inactive
18–19 June 2024	^152^Eu	8 commercial	16 h	691 pb^−1^	Inactive

**Table 3:** The resolutions obtained for one of the detectors across the different calibration runs.

Energy (keV)	FWHM in 6–7 May 2024 (%)	FWHM in 9–10 May 2024 (%)	FWHM in 31 May 2024 (%)	FWHM in 18 June 2024 (%)
40 keV	10.31 ± 0.01	10.01 ± 0.08	9.8 ± 0.4	10.51 ± 0.10
121 keV	4.18 ± 0.01	4.11 ± 0.05	4.1 ± 0.1	4.31 ± 0.05
244 keV	3.20 ± 0.03	3.15 ± 0.08	3.0 ± 1.0	3.42 ± 0.73
344 keV	3.19 ± 0.01	2.87 ± 0.04	3.4 ± 0.3	4.31 ± 0.06

## Data Availability

Dataset available on request from the authors.

## Abbreviations

The following abbreviations are used in this manuscript:

MDPI	Multidisciplinary Digital Publishing Institute
CZT, CdZnTe	Cadmium Zinc Telluride
QED	Quantum ElectroDynamics
DiFC	Dipartimento di Fisica e Chimica Emilio Segré
LNF	Laboratori Nazionali di Frascati
INFN	Istituto Nazionale di Fisica Nucleare
CSP	Charge Sensitive Preamplifier
DPP	Digital Pulse Processing
IP	Interaction Point
PMT	PhotoMultiplier Tube
ADC	Analogue to Digital Converter
FWHM	Full Width at Half Maximum
IMEM	Istituto dei Materiali per l’Elettronica ed il Magnetismo
CNR	Consiglio Nazionale delle Ricerche
